# The Single-Dose Application of Interleukin-4 Ameliorates Secondary Brain Damage in the Early Phase after Moderate Experimental Traumatic Brain Injury in Mice

**DOI:** 10.3390/ijms241612756

**Published:** 2023-08-14

**Authors:** Johannes Walter, Jannis Mende, Samuel Hutagalung, Obada T. Alhalabi, Martin Grutza, Guoli Zheng, Thomas Skutella, Andreas Unterberg, Klaus Zweckberger, Alexander Younsi

**Affiliations:** 1 Department of Neurosurgery, Heidelberg University Hospital, Im Neuenheimer Feld 400, 69120 Heidelberg, Germany; jannisme@icloud.com (J.M.); hutagalung.dra@gmail.com (S.H.); obada.alhalabi@med.uni-heidelberg.de (O.T.A.); martin.grutza@med.uni-heidelberg.de (M.G.); g.zheng@stud.uni-heidelberg.de (G.Z.); andreas.unterberg@med.uni-heidelberg.de (A.U.); k.zweckberger@klinikum-braunschweig.de (K.Z.); 2 Institute for Anatomy and Cell Biology, Heidelberg University, Im Neuenheimer Feld 307, 69120 Heidelberg, Germany; skutella@ana.uni-heidelberg.de

**Keywords:** traumatic brain injury, TBI, controlled cortical impact, CCI, interleukin-4, IL-4, inflammation, immunomodulation, secondary brain damage, behavioral test

## Abstract

Activation of the interleukin-4 (IL-4) pathway ameliorates secondary injury mechanisms after experimental traumatic brain injury (TBI); therefore, we assessed the effect of a therapeutic IL-4 administration on secondary brain damage after experimental TBI. We subjected 100 C57/Bl6 wildtype mice to controlled cortical impact (CCI) and administered IL-4 or a placebo control subcutaneously 15 min thereafter. Contusion volume (Nissl staining), neurological function (hole board, video open field, and CatWalkXT^®^), and the immune response (immunofluorescent staining) were analyzed up to 28 days post injury (dpi). Contusion volumes were significantly reduced after IL-4 treatment up to 14 dpi (e.g., 6.47 ± 0.41 mm^3^ vs. 3.80 ± 0.85 mm^3^, *p* = 0.011 3 dpi). Macrophage invasion and microglial response were significantly attenuated in the IL-4 group in the acute phase after CCI (e.g., 1.79 ± 0.15 Iba-1+/CD86+ cells/sROI vs. 1.06 ± 0.21 Iba-1/CD86+ cells/sROI, *p* = 0.030 in the penumbra 3 dpi), whereas we observed an increased neuroinflammation thereafter (e.g., mean GFAP intensity of 3296.04 ± 354.21 U vs. 6408.65 ± 999.54 U, *p* = 0.026 in the ipsilateral hippocampus 7 dpi). In terms of functional outcome, several gait parameters were improved in the acute phase following IL-4 treatment (e.g., a difference in *max intensity* of −7.58 ± 2.00 U vs. −2.71 ± 2.44 U, *p* = 0.041 3 dpi). In conclusion, the early single-dose administration of IL-4 significantly reduces secondary brain damage in the acute phase after experimental TBI in mice, which seems to be mediated by attenuation of macrophage and microglial invasion.

## 1. Introduction

Traumatic brain injury (TBI) remains one of the major global health concerns and poses a significant burden on patients, their relatives, and health care systems [1,2]. Besides acute functional impairments, such as seizures, sensorimotor deficits, or even coma, up to 30% of the patients suffering from TBI experience significant long-term symptoms [1,2,3]. However, despite TBI’s socioeconomic significance and considerable scientific efforts to develop new treatment strategies, therapeutic options remain strictly symptomatic because, to date, in contrast to promising preclinical findings, all large randomized controlled clinical trials evaluating treatments directly interfering with TBI pathophysiology have failed to prove any beneficial effect on TBI outcome [4,5,6,7].

The shortcomings of the clinical translation of experimental results are mainly due to the immense complexity of this pathophysiology. Simplified, the evolution of brain damage after TBI can be divided into a primary injury only amenable to prevention and a secondary injury, which progresses with temporal delay, making it accessible for therapeutic measures. However, a plethora of pathophysiologic mechanisms, such as the breakdown of the blood–brain barrier, capillary leakage, edema formation, ischemia, acute and chronic neuroinflammation, oxidative stress, and excitotoxicity, are involved in this secondary injury progression [8,9,10]. Additionally, these mechanisms do not act separately but rather closely interact and influence one another, further complicating the balance between possible beneficial and harmful treatment effects.

In the last decade, understanding the role of inflammatory processes in the evolution of secondary brain damage after TBI has significantly advanced, elaborating a wide range of new possible treatment targets [9,10,11,12,13]. Traditionally, treatment approaches modulating the immune system have focused on directly antagonizing pro-inflammatory processes; yet, despite promising preclinical findings, none of the treatments have proven beneficial in large-scale clinical trials [14,15,16,17,18,19]. However, in recent years, treatment approaches utilizing the body’s anti-inflammatory mechanisms to achieve a more physiologic anti-inflammatory response have gained popularity in treating acute neurotrauma and have generated encouraging preclinical results [20,21,22,23,24,25].

To this end, in a recent study, we could show that the anti-inflammatory cytokine, interleukin-4 (IL-4), which is mainly expressed by M2-polarized microglia and macrophages and has proven to mediate significant neuroprotective effects both, in vitro and in various in vivo models of acute neuronal injury, has substantial neuroprotective potential in the context of experimental TBI as well and thus, poses an intriguing novel treatment option [26,27,28,29,30]. Despite the promising results of our previous study, data on the therapeutical application of IL-4 to ameliorate the sequelae of TBI are scarce. Only three studies have evaluated a therapeutic use of IL-4 in the context of experimental TBI; however, in all three studies, extensive treatment regimens over up to four weeks after trauma induction have been used and outcome assessment has been focused on chronic timepoints after TBI induction [23,24,25]. Yet, the effects of an IL-4 treatment on the acute and subacute phases are of great interest to fully assess the possible neuroprotective potential of the substance. In addition, shorter treatment regimens would be more feasible in a clinical setting and consequently, could facilitate clinical translation.

To gain more detailed insight into this topic, we hypothesized that a therapeutic administration of IL-4 significantly reduces secondary brain damage within the first four weeks after experimental TBI in mice. To test this hypothesis, we utilized a pragmatic approach and evaluated the effect of an early, single-dose application of IL-4 on lesion volumes, the local inflammatory response in the brain, and functional outcome parameters up to 28 days after trauma induction, starting on the first day after TBI.

## 2. Results

### 2.1. Histological Assessment

#### 2.1.1. Lesion Volume

**Figure 1:**
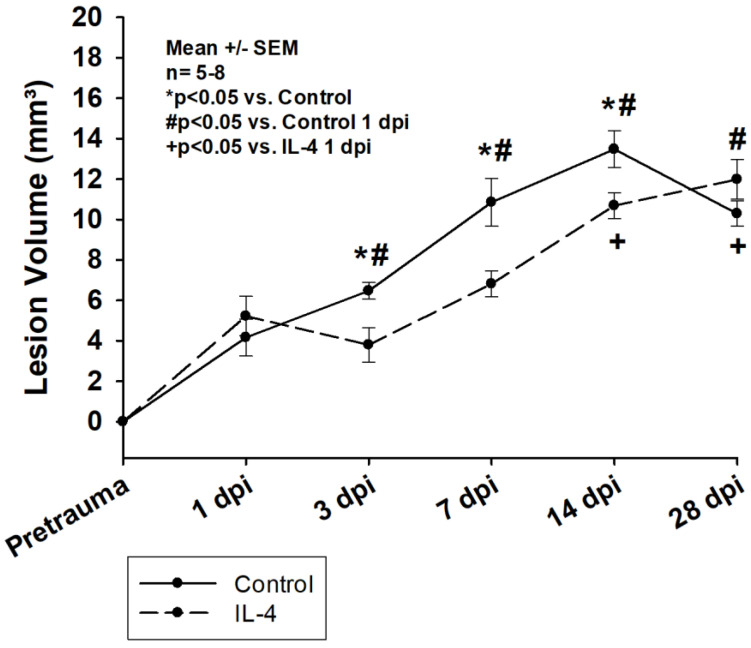
Lesion volume determined after Nissl staining of the brain sections within the first four weeks after CCI. The exact number of animals analyzed per group are summarized in Supplement Table S1. Normal distribution was verified by Shapiro–Wilk test, and Student’s *t*-test was performed for comparison of IL-4 and control groups at the respective timepoints. Statistical significance was assumed for *p* < 0.05. * *p* < 0.05 vs. control, # *p* < 0.05 vs. control 1 dpi, + *p* < 0.05 vs. IL-4 1 dpi. SEM: standard error of the mean, IL-4: interleukin-4 treated mice, dpi: days post injury.

Lesion volumes significantly increased within the first two weeks after CCI in both groups and remained stable thereafter (e.g., 5.22 ± 0.98 mm^3^ 1 dpi vs. 10.69 ± 0.64 mm^3^ 14 dpi, *p* < 0.001 for IL-4 and 4.16 ± 0.91 mm^3^ 1 dpi vs. 13.48 ± 0.90 mm^3^ 14 dpi, *p* < 0.001 for control, Figure 1). The application of IL-4 resulted in a statistically significant reduction in lesion volume 3 dpi (6.47 ± 0.41 mm^3^ vs. 3.80 ± 0.85 mm^3^, *p* = 0.011 for control and IL-4, respectively), 7 dpi (10.85 ± 1.19 mm^3^ vs. 6.82 ± 0.65 mm^3^, *p* = 0.020 for control and IL-4, respectively), and 14 dpi (13.48 ± 0.90 mm^3^ vs. 10.69 ± 0.64 mm^3^, *p* = 0.024 for control and IL-4, respectively).

#### 2.1.2. Macrophage Invasion

**Figure 2:**
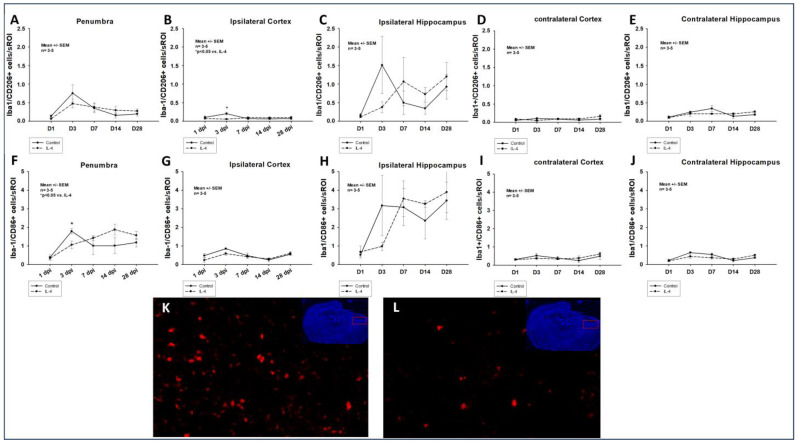
Number of M2-polarized Iba-1/CD206+ (**A**–**E**) and M1-polarized Iba-1/CD86+ (**F**–**J**) macrophages in the traumatic penumbra (**A**–**F**), the ipsilateral cortex (**B**,**G**), the ipsilateral hippocampus (**C**,**H**), the contralateral cortex (**D**,**I**), and the contralateral hippocampus (**E**,**J**) within the first four weeks after CCI. Panels (**K**) (control) and (**L**) (IL-4 treated animal) illustrate examples of CD86 stainings in the penumbra 3 dpi (images obtained using Zeiss Plan-Apochromat 20×/0.8 M27 objective, Zeiss, Oberkochen, Germany). The exact number of animals analyzed per group are summarized in Supplement Table S1. Normal distribution was verified by Shapiro–Wilk test, and Student’s *t*-test was performed for comparison of IL-4 and control groups at the respective timepoints. Statistical significance was assumed for *p* < 0.05. * *p* < 0.05 vs. IL-4. SEM: standard error of the mean, IL-4: interleukin-4 treated mice, sROI: standardized region of interest, dpi: days post injury.

The application of IL-4 significantly reduced the number of both M1-polarized, Iba-1/CD86+ and M2-polarized, Iba-1/CD206+ macrophages in the penumbra as well as in the ipsilateral cortex in the early phase after CCI (e.g., 1.79 ± 0.15 Iba-1/CD86+ cells/sROI vs. 1.06 ± 0.21 Iba-1/CD86+ cells/sROI, *p* = 0.030 in the penumbra 3 dpi for control and IL-4, respectively). However, after initial attenuation of macrophage invasion, a non-significant increase in the numbers of macrophages could be detected in the penumbra of IL-4 treated animals 14 dpi and 28 dpi. Of note, the invasion of both M1- and M2-polarized macrophages was significantly more pronounced in the ipsilateral hippocampi compared to the ipsilateral cortex (e.g., 0.85 ± 0.04 vs. 1.79 ± 0.15 Iba-1/CD86+ cells/sROI, *p* = 0.029 and 0.20 ± 0.04 vs. 1.51 ± 0.78 Iba-1/CD206+ cells/sROI, *p* = 0.029 3 dpi; Figure 2).

#### 2.1.3. Astroglial Response

**Figure 3:**
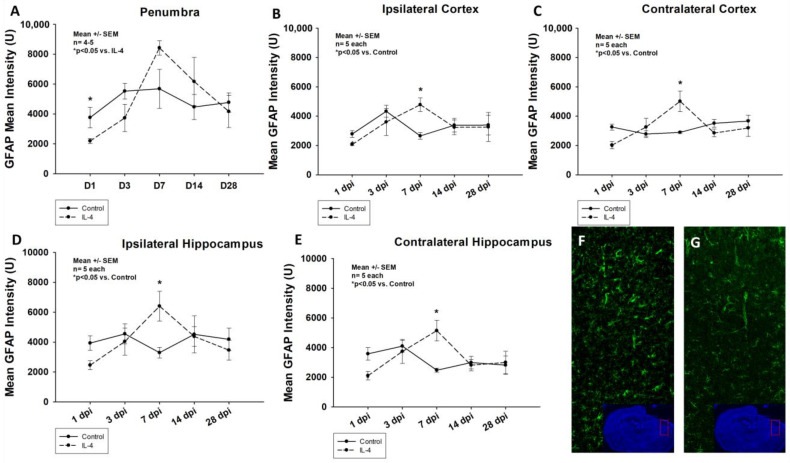
Mean GFAP intensity in the penumbra (panel (**A**)), ipsi- and contralateral cortices (**B**,**C**) as well as ipsi- and contralateral hippocampi (**D**,**E**) within the first four weeks after CCI. Panels (**F**) (control) and (**G**) (IL-4 treated animal) illustrate examples of GFAP stainings in the penumbra 1 dpi (images obtained using Zeiss Plan-Apochromat 20×/0.8 M27 objective, Zeiss, Oberkochen, Germany). The exact number of animals analyzed per group are summarized in Supplement Table S1. Normal distribution was verified by Shapiro–Wilk test, and Student’s *t*-test was performed for comparison of IL-4 and control groups at the respective timepoints. Statistical significance was assumed for *p* < 0.05. * *p* < 0.05 vs. control. SEM: standard error of the mean, IL-4: interleukin-4 treated mice, GFAP: glial fibrillary acidic protein, dpi: days post injury.

A similar effect of the administration of IL-4 on the astroglial response after CCI, measured by quantifying the GFAP intensity, could be detected throughout all sROIs. After an initial significant reduction (e.g., mean GFAP intensity of 3760.18 ± 695.24 U vs. 2191.76 ± 165.83 U, *p* = 0.008 in the penumbra 1 dpi for control and IL-4, respectively), a statistically significant increase in GFAP intensity could be detected 7 dpi (e.g., mean GFAP intensity of 3296.04 ± 354.21 U vs. 6408.65 ± 999.54 U, *p* = 0.026 in the ipsilateral hippocampus for control and IL-4, respectively). Thereafter, there were no differences between the two groups in terms of astroglial reaction (Figure 3).

#### 2.1.4. Neuronal Apoptosis

No significant difference in neuronal apoptosis, characterized by the presence of NeuN/TUNEL+ cells, could be found in the penumbra as well as the contralateral cortex and hippocampus of IL-4 treated animals compared to control animals throughout the experiment (Supplement Figure S1). Interestingly, the early single-dose application of IL-4 resulted in a significantly increased rate of neuronal apoptosis in the ipsilateral cortex 14 dpi (1.40 ± 0.41 vs. 6.23 ± 1.46 NeuN/TUNEL+ cells/sROI, *p* = 0.010 for control and IL-4), while in the ipsilateral hippocampus, the number of NeuN/TUNEL+ cells was significantly reduced in the IL-4 group 3 dpi (1.17 ± 0.28 vs. 0.41 ± 0.07 NeuN/TUNEL+ cells/sROI, *p* = 0.028 for control and IL-4).

#### 2.1.5. Myelinization

When assessing the MBP intensity as a surrogate for myelinization in the ipsilateral ROIs, there were no differences between the IL-4 and control animals at any timepoint. Interestingly though, in the contralateral cortex, an early increase in MBP intensity after IL-4 treatment could be observed, followed by a decrease in myelinization in the more chronic phase of the experiment, reaching statistical significance 28 dpi (mean MBP intensity of 5649.40 ± 465.24 U vs. 4383.55 ± 267.99 U, *p* = 0.046 for control and IL-4, respectively; Supplement Figure S2).

#### 2.1.6. Oligodendroglial Regeneration

Olig^2+^ cells were significantly increased in IL-4 treated animals in the contralateral cortex 7 dpi (9.44 ± 1.21 Olig^2+^ cells vs. 6.68 ± 0.22 Olig^2+^ cells, *p* = 0.04); however, IL-4 treatment did not have any effect on oligodendroglial regeneration in the other ROIs at any timepoint (Supplement Figure S3).

### 2.2. Functional Outcome

#### 2.2.1. Body Weight

For the first week after CCI, animals of both groups lost a significant amount of body weight (e.g., −6.8% ± 0.5% and −6.5% ± 0.5%, *p* < 0.001 for control and IL-4 treated animals 1 dpi, respectively), while returning to baseline by 14 dpi and starting to gain weight again thereafter. The administration of IL-4 did not have any effect on posttraumatic weight loss (Supplement Figure S4).

#### 2.2.2. Hole Board

**Figure 4:**
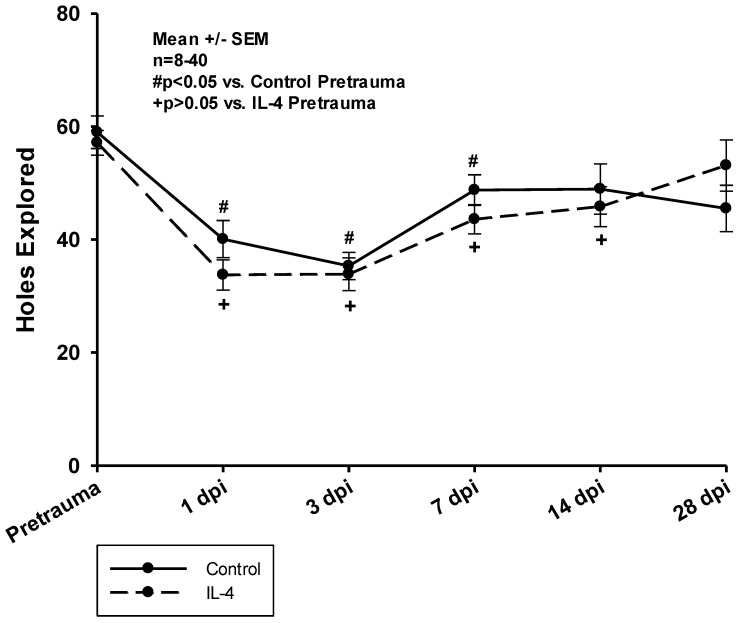
Total number of holes explored on the hole board within a test period of ten minutes. The exact number of animals analyzed per group are summarized in Supplement Table S1. Normal distribution was verified by Shapiro–Wilk test, and Student’s *t*-test was performed for comparison of IL-4 and control groups at the respective timepoints. Statistical significance was assumed for *p* < 0.05. # *p* < 0.05 vs. control pretrauma, + *p* < 0.05 vs. IL-4 pretrauma. SEM: standard error of the mean, IL-4: interleukin-4 treated mice, dpi: days post injury.

In both groups, animals explored significantly less holes within the test period of ten minutes in the first week after CCI. Control animals returned to baseline performance slightly earlier than IL-4 treated animals (14 dpi vs. 28 dpi); however, there were no difference between the groups concerning the total number of hole board explorations at any timepoint (Figure 4).

#### 2.2.3. Video Open Field

**Figure 5:**
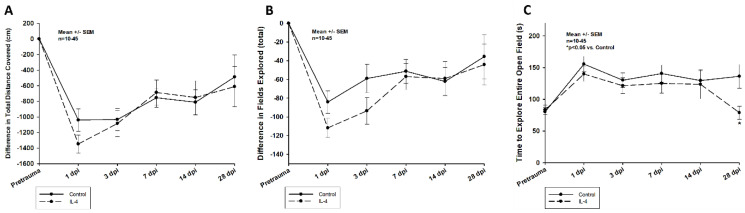
Difference in total distance covered (**A**), in total number of fields explored (**B**), and time to explore the entire open field (**C**) within the first four weeks after CCI. The exact number of animals analyzed per group are summarized in Supplement Table S1. Normal distribution was verified by Shapiro–Wilk test, and Student’s *t*-test was performed for comparison of IL-4 and control groups at the respective timepoints. Statistical significance was assumed for *p* < 0.05. * *p* < 0.05 vs. control. SEM: standard error of the mean, IL-4: interleukin-4 treated mice, dpi: days post injury.

The animals of both groups were impaired in their exploration behavior throughout the entire observation period (Figure 5). Il-4 treated animals tended to be more affected within the first week after CCI; yet, the differences were not statistically significant. In the more chronic phase of the experiment, it took IL-4 treated animals significantly less time to explore the entire open field compared to animals of the control group (136.29 ± 18.70 s vs. 78.91 ± 10.44 s, *p* = 0.016 for control and IL-4 28 dpi, respectively).

#### 2.2.4. CatWalkXT^®^

**Figure 6:**
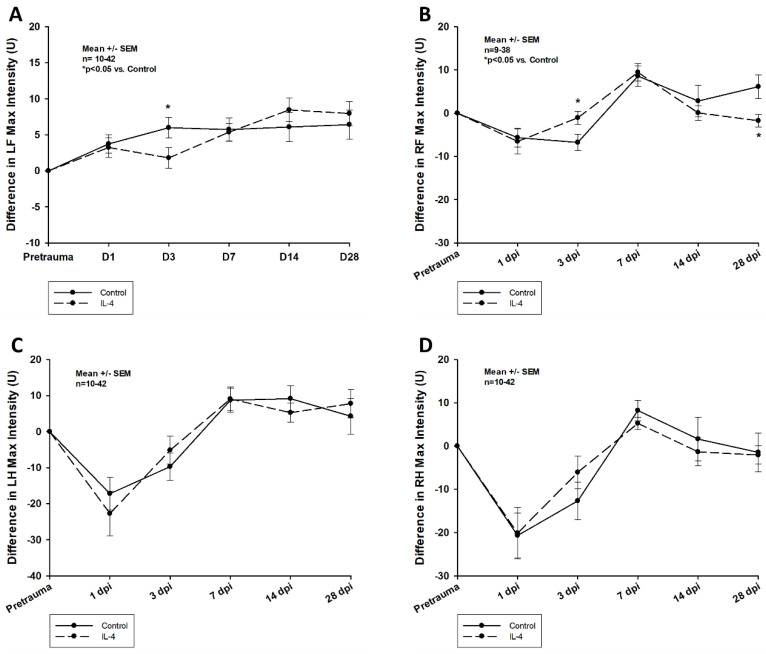
Difference in *max intensity* in the left frontpaws (**A**), right frontpaws (**B**), left hindpaws (**C**), and right hindpaws (**D**) within the first four weeks after CCI. The exact number of animals analyzed per group are summarized in Supplement Table S1. After multiple group comparisons of all neurotest timepoints by Kruskal–Wallis ANOVA on ranks test, Dunn’s test was performed to isolate the group or groups that significantly differed from the others. Statistical significance was assumed for *p* < 0.05. * *p* < 0.05 vs. control. SEM: standard error of the mean, IL-4: interleukin-4 treated mice, LF: left frontpaw, RF: right frontpaw, LH: left hindpaw, RH: right hindpaw, dpi: days post injury.

**Figure 7:**
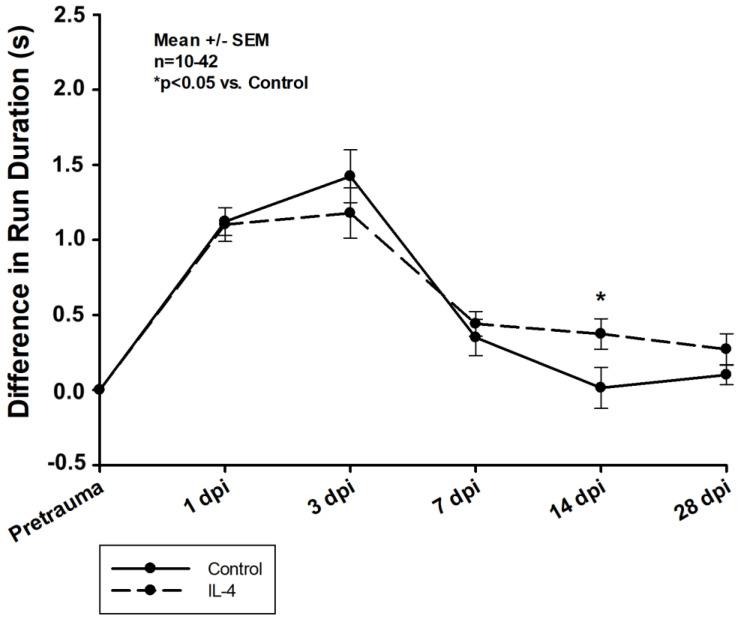
Difference in *run duration* within the first four weeks after CCI. The exact number of animals analyzed per group are summarized in Supplement Table S1. After multiple group comparisons of all neurotest timepoints by Kruskal–Wallis ANOVA on ranks test, Dunn’s test was performed to isolate the group or groups that significantly differed from the others. Statistical significance was assumed for *p* < 0.05. * *p* < 0.05 vs. control. SEM: standard error of the mean, IL-4: interleukin-4 treated mice, dpi: days post injury.

The administration of IL-4 affected posttraumatic gait impairments in two distinct patterns:

Firstly, early recovery of the static single paw parameter *max intensity* was significantly improved after IL-4 treatment in the right frontpaws (difference in *max intensity* of −7.58 ± 2.00 U vs. −2.71 ± 2.44 U, *p* = 0.041 for control and IL-4 3 dpi, respectively). However, its long-term recovery was reduced (difference in *max intensity* of 6.14 ± 2.70 U vs. −1.75 ± 1.46 U, *p* = 0.037 for control and IL-4 28 dpi, respectively; Figure 6). Secondly, IL-4 treatment did not have any beneficial effect on the dynamic CatWalkXT^®^ parameter *run duration* in the acute phase after CCI, while in contrast, its impairment recovered even slower in IL-4 treated animals 14 dpi (difference in *run duration* of 0.01 ± 0.14 s vs. 0.37 ± 0.10 s, *p* = 0.044 for control and IL-4, respectively; Figure 7).

## 3. Discussion

### 3.1. The Early Single-Dose Application of IL-4 Leads to a Significant Reduction in Lesion Volume

In our study, lesion volumes were similar to the ones observed in other studies evaluating possible IL-4 treatment effects ([24]). We show that the early single-dose administration of IL-4 leads to a significant reduction in lesion volume in the acute and subacute stages after CCI (Figure 1). Interestingly, the rate of secondary lesion expansion was very similar in both groups between 3 dpi and 14 dpi, possibly indicating that the thereapplication of IL-4 had an early beneficial effect on processes involved in secondary lesion expansion. However, the treatment might only have postponed these processes, which in return, resulted in a prolonged progression of the lesion in IL-4 treated animals compared to the control group and ultimately, in a similar lesion size 28 dpi. Of note, in previous experiments using IL-4 knockout mice, we could show that a complete knockout of IL-4 resulted in a significant reduction of the lesion volume only in the acute phase after CCI, while there were no difference in lesion volumes compared to wildtype mice 7 dpi. [26] These findings might indicate that the difference in IL-4 bioactivity between animals receiving exogenous IL-4 and wildtype (control) animals is more relevant than the one between wildtype and knockout animals and therefore, that knockout experiments, while extremely important in the early stages of assessing new treatment concepts, might have relevant limitations in predicting possible treatment effects in wildtype animals.

When assessing the spatial profile of neuronal loss after CCI, we observed that the effect of IL-4 on lesion volume might be based on a reduced neuronal loss in the ipsilateral cortex rather than in the direct traumatic penumbra (Supplement Figure S1). A possible explanation could be that the neurons in close proximity to the primary injury site are damaged too severely and their microenvironment is altered too extensively immediately after CCI, leaving them in a non-salvageable state, whereas on the other hand, neurons located further away could possibly still recover.

### 3.2. The Neuroprotective Effect of IL-4 Seems to Be Mediated by an Attenuation of the Inflammatory Response

In our study, we observed an IL-4 induced, significant reduction of both Iba-1/CD86+, M1-polarized and Iba-1/CD206+, M2-polarized macrophages in the traumatic penumbra as well as the ipsilateral cortex 3 dpi (Figure 2). Therefore, the observed neuroprotective effect of IL-4 could be mediated by an attenuated cellular inflammatory response following CCI. This is further supported by the fact that the astroglial response was also significantly ameliorated in IL-4 treated animals in the acute phase after CCI (Figure 3).

It is well described that macrophages and microglia do not proliferate within the first 24 to 48 h after TBI, which is well in line with our observations (e.g., peak numbers of M1/M2 macrophages 3 dpi, Figure 2) [11]. This might explain that despite the short half-life of IL-4, we detected the first treatment effect of IL-4 on macrophage invasion only on day three after CCI. Following the initial attenuation of reactive astrocytosis and macrophage invasion in the acute phase after CCI, we observed a rebound effect with an excessive cellular immune response, which might possibly be explained by the fact that IL-4 is rapidly cleared by renal excretion, resulting in a half-life of only 20 min and therefore, limiting the therapeutic potential of a single-dose application [31].

Enam et al. have reported on an IL-4 induced shift in macrophage polarization towards the anti-inflammatory M2-type after CCI; however, we could not detect such an effect in our study [25]. A possible explanation could be that Enam et al. started their treatment on day five after CCI, while in our study, due to its short half-life, IL-4 most likely did not have any direct treatment effect at this timepoint any more. Furthermore, in the study by Enam et al., IL-4 was administered via genetically altered mesenchymal stem cells, and therefore, a continuous drug application was established, which most likely led to higher and more stable IL-4 concentrations than in our study. These observations possibly indicate a time and dose dependency of the effect of IL-4 on macrophage polarization after CCI.

### 3.3. The Single Dose IL-4 Treatment Did Not Improve Oligodendroglial Regeneration or Myelinization

In contrast to previous reports by Pu et al., we did not observe any effect of IL-4 treatment on the presence of Olig^2+^ oligodendrocytes after CCI and thus, possible oligodendroglial regeneration [24]. This is most likely due to the fact that Pu et al. applied a repetitive dosing scheme and a higher IL-4 dose compared to the single-dose application in our study, resulting in a longer lasting and more pronounced treatment effect. Similarly, myelinization was not positively affected by the IL-4 treatment, correlating with our findings on the number of oligodendrocytes.

### 3.4. The Effect of IL-4 on Structural Damage Is Greater Than on Neurological Function

In our study, we could show that an early single-dose administration of IL-4 reduces lesion volumes up to 14 dpi; however, the beneficial effects on neurological function were only detectable in the acute phase after CCI, while in the chronic phase, IL-4 treatment possibly even slowed down neurological recovery (Figure 1 and Figure 6). This again demonstrates that neurological function does not necessarily correlate to lesion volume alone, an observation that has been reported before [32]. Furthermore, temporal profiles of the effects of IL-4 on the inflammatory response (early attenuation followed by a rebound with increased inflammation) and neurological function (initial improvement with delayed recovery in the chronic phase) were very similar, indicating that attenuating the inflammatory response might be more important than focusing on solely reducing lesion size in order to improve neurological recovery after experimental TBI. Of note, several studies have reported on long-term beneficial effects of IL-4 treatment in CCI models, and in our previous experiments using IL-4 knockout mice, we also observed beneficial effects of IL-4 on neurological function for up to a week after CCI [23,24,25,26]. The discrepancy to the observations of our current study are most likely explained by the fact that in all IL-4 treatment studies, IL-4 was administered utilizing repetitive dosing regimens or a continuous application, leading to higher doses of IL-4 for a longer period of time, while a genetic knockout of IL-4 obviously results in a permanent lack of IL-4 and should, therefore, result in the most profound impairments of neurological function. Yet, even though it would be of great interest to quantify the full potential of an IL-4 treatment, there are no available data on the effects of an IL-4 knockout on functional impairments in the chronic phase after CCI, prohibiting the comparison of our current study results.

### 3.5. Limitations

In our study we systemically administered IL-4 already 15 min after trauma induction. Despite secondary injury processes after traumatic brain injury being slightly accelerated in rodents compared to humans, this is still a very early timepoint limiting the translation of our observations into a clinical context. Therefore, further studies need to assess the therapeutic window of IL-4 after experimental TBI. Furthermore, we only administered a single dose of IL-4, which potentially lead to a rebound effect. Repetitive dosing or serum half-life extension, e.g., by polymer conjugation, need to be evaluated as options to avoid such a rebound effect and therefore, might improve neurological recovery in the chronic phase after TBI as well. Moreover, we did not assess the effect of IL-4 on the systemic inflammatory response, which might also influence neurological recovery. In addition, the discrepancy between the robust effect of IL-4 treatment on lesion volume and the moderate effects on neurological recovery again illustrate the difficulty of detecting subtle changes in neurological function after experimental TBI in rodents. Moreover, the number of datapoints are limited, especially for some immunohistochemical analyses, which might have led to an underestimation of treatment effects. Finally, we did not screen for any possible adverse effects of IL-4 on other organ systems, which might limit its application in a clinical setting.

## 4. Materials and Methods

### 4.1. Animals

A total of 100 male C57Bl/6 wildtype mice (Janvier Labs, Le Genest Saint Isle, France) with a body weight of 20–25 g aged six to eight weeks were used for the experiments. The number of animals per group was calculated to detect a 30% difference in the primary outcome parameter, the lesion volume with a power of 0.8, assuming a standard deviation of 0.2. The animals were assigned to the respective experimental groups (one-, three-, seven-, and 28-day groups with either the control or IL-4 treatment for each timepoint, resulting in a total of eight experimental groups; Supplemental Table S1) by drawing lots. All surgeons and outcome assessors were blinded to the treatment; unblinding was performed after final data analysis. The animals were kept under a 12 h-day/12 h-night cycle; food and water were accessible ad libitum. Body weight was measured daily, and the relative weight loss (difference in pre- and postoperative body weight divided by preoperative body weight) was documented. All procedures were approved by the Animal Care Committee of the federal government (Regierungspräsidium Karlsruhe, approval number G-296/19). Postoperative health screens and hygiene management checks were performed according to the Federation of European Laboratory Animal Science Associations guidelines and recommendations [33].

### 4.2. Controlled Cortical Impact (CCI)

The trauma was induced using the CCI model as described before [26,32,34,35,36,37,38,39,40,41]. In brief, the animals were placed on a heating pad adjusted to 37°C to prevent intraoperative hypothermia and the head was fixed in a stereotactic frame (Kopf Instruments, Tujunga, CA, USA) after establishing sufficient analgesia (buprenorphine (Indivior Europe Ltd., Dublin, UK), 0.1 mg/kg s.c. 30 min prior to surgery, and carprofen (Norbrook Laboratories, Newry, Northern Ireland), 5 mg/kg s.c. 12 and 24 h after CCI)) and sedation (Isoflurane/oxygen/air (1%/30%/69%)). After midline skin incision, a right parietal craniotomy was performed microsurgically, creating a bone flap hinged on the superior sagittal sinus. Then, the bone flap was retracted medially leaving the dura mater intact. After positioning the impactor tip (2 mm in diameter) directly on the dura mater, a standardized trauma was induced using the following parameters: impact depth 1 mm, dwell time 150 ms, impactor speed 8 m/s. Directly after trauma induction, the bone flap was quickly repositioned and sealed using histoacrylic glue (B. Braun Melsungen AG, Melsungen, Germany, 1050044). The skin was closed using interrupted sutures, and the mice were put back into their cages after terminating sedation. Postoperatively, the cages were put on a heating pad at 34°C for one hour to prevent hypothermia.

### 4.3. Drug Administration

After randomized assignment to the respective experimental group, either IL-4 (CYT-282, ProSpec, St. Louis, MO, USA) in a concentration of 5 µg/kg (diluted in phosphate buffered saline (PBS) to 1 µg/mL, resulting in an injection volume of 100–125 µL per injection), or the respective amount of PBS was administered subcutaneously 15 min after trauma induction.

### 4.4. Histological Assessment

After sacrificing the animals, brains were removed and shock frozen on dry ice immediately. Using a cryostat (Leica CM3050S, Leica Biosystems, Oberkochen, Germany) coronal sections of 10 µm in thickness were prepared every 300 µm, starting 1000 µm behind the olfactory bulb.

### 4.5. Determination of Lesion Size

The frozen brain sections were stained according to the Nissl protocol. Images of each section were taken using a computer-mounted camera (LSM 700; Carl-Zeiss, Oberkochen, Germany, Supplement Figure S1). Lesion areas were determined for each section using ImageJ (National Institute of Health, Bethesda, MD, USA), and lesion volumes (Vn) were calculated using the following formula:

Vn = (A1 + A2 + … + Ax) × 0.3

### 4.6. Immunofluorescent Staining

The frozen brain sections were blocked with a blocking solution containing 0.3% PB-Tween and 2% bovine serum albumin (BSA, all Sigma-Aldrich, St. Louis, MO, USA) for 30 min at room temperature. The following primary antibodies, diluted in the same blocking solution, were then added and incubated at 4 °C overnight: anti-Iba-1 (1:500, FUJIFILM Wako Pure Chemical Corporation, Osaka, Japan, 019-19741), anti-CD86 (1:200, Thermo Fisher, Waltham, MA, USA, 14-0862-81), anti-CD206 (1:200, R&D Systems, Minneapolis, MN, USA, AF2535), anti-GFAP (1:200, Abcam, Boston, MA, USA, ab16667), anti-MBP (1:100, Abcam, ab218011), anti- Olig2 (1:50, R&D Systems, AF2418), and anti-NeuN (1:200, Synaptic Systems, Göttingen, Germany, 266 004).

After removal of the primary antibody dilution, the secondary antibodies (anti-rabbit 488, 1:1000, Sigma-Aldrich, SAB4600234; anti-goat 546, 1:1000, Invitrogen, A-11056; anti-goat 488, 1:1000, Thermo Fisher, A11055; anti-guinea pig 546, 1:500, Thermo Fisher, A11074), diluted in blocking solution without BSA were applied for 1 h at room temperature. DAPI (1: 10,000; Sigma-Aldrich, St. Louis, MO, USA, A11055) was added for 10 min before the cells/tissue sections were subjected to imaging analysis.

For assessment of apoptosis, a terminal deoxynucleotidyl transferase dUTP nick-end-labeling (TUNEL) assay (Thermo Fisher, C10245) was applied according to manufacturer guidelines.

### 4.7. Imaging Analysis

All images were obtained using a confocal laser scanning microscope (LSM 700, Carl-Zeiss, Germany) at 20× magnification. Using ImageJ, the five regions of interest (ROI), ipsi- and contralateral hippocampus, ipsi- and contralateral cortex, and traumatic penumbra, (Supplement Figure S5) were cut out from a coronal section at the level of the hippocampus and the images split into single channels. For quantitative analysis of M1- and M2-polarized macrophage invasion (Iba-1/CD86+ and Iba-1/CD206+ cells, respectively), neuronal apoptosis (NeuN/TUNEL+ cells) and oligodendroglial regeneration (Olig^2+^ cells), a two-step approach was utilized. In the first step, the machine-learning based ilastik tool was manually trained to detect colocalization of DAPI and the respective fluorescent antibody (cells) and exclude fluorescence signals without DAPI colocalization (background noise). Then, the automated cell detection was manually verified after applying the algorithm to images that had not been used for training purposes. After achieving a sufficient accuracy, the algorithm was applied to the images of the ROIs for automated cell counting, generating a greyscale probability map estimating the probability that the pixel belonged to a cell [42].

In the second step, after Gaussian blurring to further reduce background noise and applying the Otsu algorithm, the probability maps were translated into binary images using ImageJ. For automated cell counting, the binary images of the respective antibody were fused and anatomically non-colocalized signals excluded, leaving only Iba-1/CD86+, Iba-1/CD206+, and NeuN/TUNEL+ colocalized cells. The total number of cells is reported as cells per standardized region of interest (sROI), which consists of 100,000 pixels (0.04 mm^2^).

In addition to myelinization (MBP), the postraumatic astroglial response (GFAP) was assessed quantitatively by analyzing the immunointensity of the respective marker as single cell counting of GFAP+ cells was not feasible due to significant interference of individual signals. The immunointensity of the respective marker was quantified in each ROI using the ImageJ “measure” function to analyze the respective mean density (immunointensity). Intensity values are reported as mean intensity per pixel.

### 4.8. Functional Assessment

All functional tests were carried out one day prior to (baseline) as well as 1 dpi, 3 dpi, 7 dpi, 14 dpi, and 28 dpi. All animals were habituated to the new environment for one week prior to preoperative baseline assessment.

#### 4.8.1. Hole Board (HB)

The HB (Ugo Basile, Como, Italy) has been frequently used in the CCI model before and assesses exploration behavior and locomotion [26,36,43,44,45]. The animals are placed in the middle of a square-shaped, elevated plate, which is perforated with 16 holes, and they explore the plate for ten minutes. A computer automatically records the total number of explored holes within the observation period.

#### 4.8.2. Video Open Field

As the HB, the video open field test assesses locomotion and exploration behavior. It has been routinely used in multiple preclinical rodent models, including the CCI model [26,46,47]. To conduct the test, the mouse is placed in the corner of a squared box and explores the area for five minutes. A camera (Burg-Guard, Meinerzhagen, Germany) mounted above the open field tracks the animal’s movements. A specialized software (SYGNIS Tracker V4.14, EXPEDEON, Heidelberg, Germany) virtually subdivides the open field area into 16 smaller fields and automatically assesses different parameters, e.g., the total number of fields visited within the observation period.

#### 4.8.3. CatWalkXT^®^

The CatWalkXT^®^ (Noldus Information Technology, Wageningen, The Netherlands) is a tool specially developed for automated, observer independent gait analysis in rodents. As the other functional tests utilized in our study, it has been widely used in various preclinical models and has been shown to generate valid data on gait and motor function in the CCI model in mice as well [36,48,49]. To conduct the CatWalkXT^®^ analysis, the mouse is placed on one side of a horizontal glass plate, which is 1.3 m in length and covered by a removable tunnel. A high-resolution camera is mounted underneath the glass plate and automatically captures the animals’ paw prints as the mouse walks across the glass plate voluntarily. The data are then automatically analyzed by the CatWalkXT^®^ software (version 10.6), and the results are reviewed manually and corrected, if necessary. In our current study, three uninterrupted runs were captured per animal, and a maximum speed variation of 60% was accepted.

The CatWalkXT^®^ software can analyze more than 100 different parameters; however, for the current experiment, we have focused on *max intensity*, a static single paw parameter assessing the maximum intensity a paw is put on the glass floor, and *run duration*, a dynamic parameter assessing the average time it takes the mouse to finish a run over the entire glass plate.

### 4.9. Data Analysis

Statistical analysis was performed using a data analysis software package (SigmaStat 12.0, Jandel Scientific, Erkrath, Germany). Data of the CatWalkXT^®^ analysis are presented as means of the difference between postoperative and preoperative results ± standard error of the mean (SEM). HB and VOF results as well as lesion volumes, cell counts, and intensities are presented as means ± SEM. Unplausible outliers (single values above Q3 + 1.5*IQR or below Q1−1.5*IQR where Q1 is the first and Q3 is the third quartile of values and IQR is the interquartile range) were excluded from data analysis. For comparisons between two groups at one single timepoint and normally distributed variables as verified by Shapiro–Wilk test, Student’s *t*-test was performed. For multiple group comparisons between all neurotest timepoints, Kruskal–Wallis ANOVA on ranks was used. If a statistically significant difference between groups was present as assessed by Kruskal–Wallis ANOVA on ranks, Dunn’s test was performed to isolate the group or groups that differed from the others. Statistical significance was assumed for *p* < 0.05.

## 5. Conclusions

The early systemic single-dose administration of IL-4 results in a significantly reduced lesion volume up to 14 days after experimental TBI in mice. This effect seems to be mediated by an attenuation of macrophage invasion and astrogliosis in the acute phase after CCI; however, possibly due to the short half-life of the substance, we observed a potential rebound effect. Further studies need to elucidate the optimal treatment regimen and therapeutic window of IL-4 to exploit its full neuroprotective potential and aid the clinical translation of preclinical results.

## Data Availability

Data supporting the reported results will be made available upon request by the corresponding authors.

## Supplementary Materials

The supporting information can be downloaded at: https://www.mdpi.com/article/10.3390/ijms241612756/s1.
