# Supplementary material for: The Single-Dose Application of Interleukin-4 Ameliorates Secondary Brain Damage in the Early Phase after Moderate Experimental Traumatic Brain Injury in Mice
Source: Int J Mol Sci. 2023 Aug 14;24(16):12756. doi: 10.3390/ijms241612756 (PMC10454634; doi:10.3390/ijms241612756)
Supplement: Supplementary file 1 [file ijms-24-12756-s001.zip › ijms-2484893-supplementary.pdf]

International Journal of
Molecular Sciences

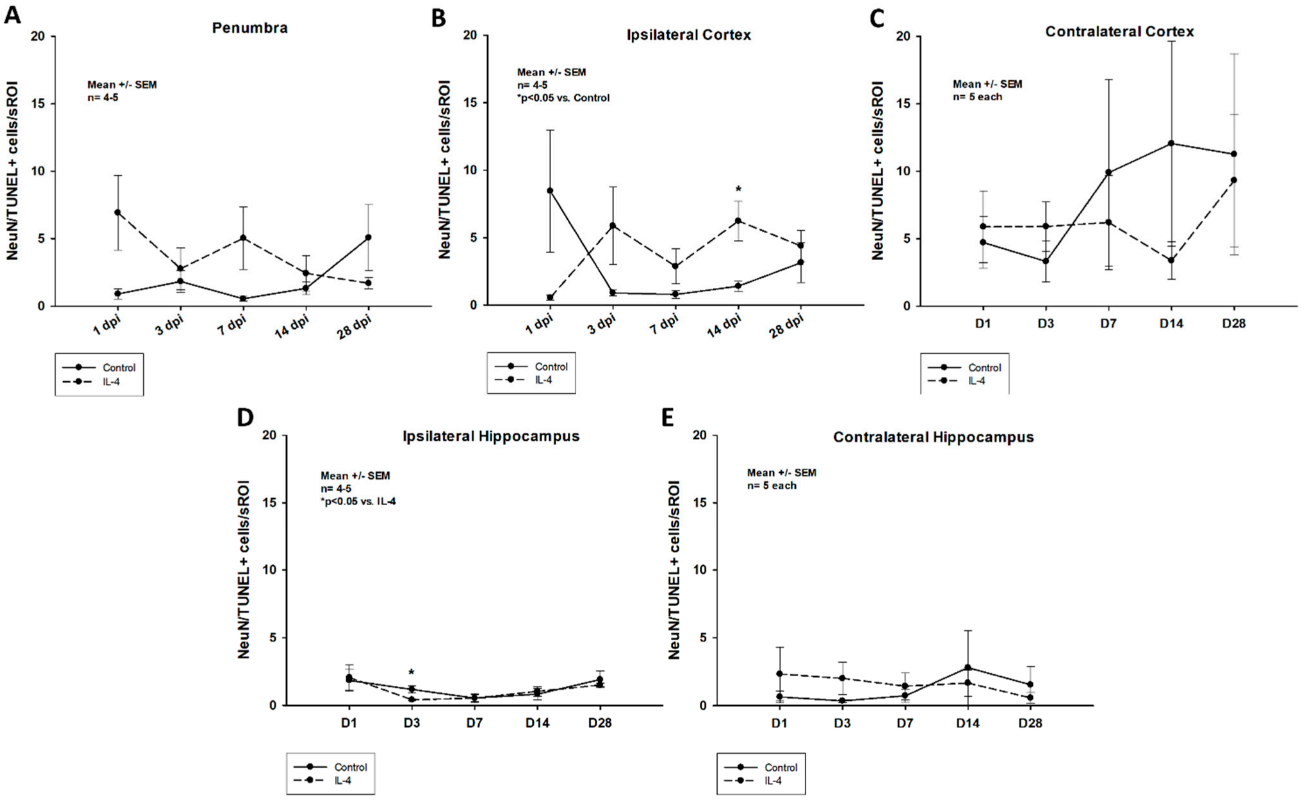

Supplement Figure S1. Number of NeuN/TUNEL+ cells in the penumbra (panel A), ipsi- and contralateral cortices (panels B and C) as well as ipsi- and contralateral hippocampi (panels D and E) within the first four weeks after CCI. The exact number of animals analyzed per group are summarized in Supplement Table S1. SEM: standard error of the mean, IL-4: interleukin-4 treated mice, TUNEL: dUTP nick end labeling, sROI: standardized region of interest, dpi: days post injury.

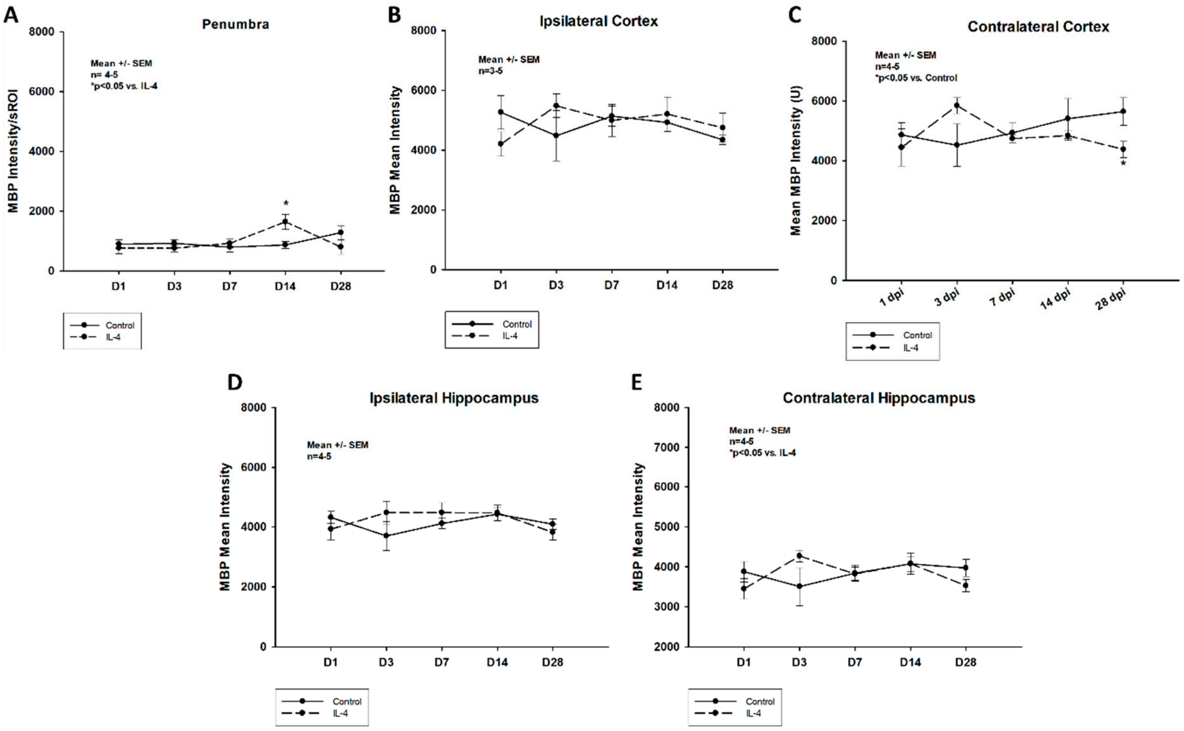

Supplement Figure S2. Mean MBP intensity as a surrogate for myelinization in the penumbra (panel A), ipsi- and contralateral cortices (panels B and C) as well as ipsi- and contralateral hippocampi (panels D and E) within the first four weeks after CCI. The exact number of animals analyzed per group are summarized in Supplement Table S1. SEM: standard error of the mean, IL-4: interleukin-4 treated mice, MBP: myelin basic protein, dpi: days post injury.

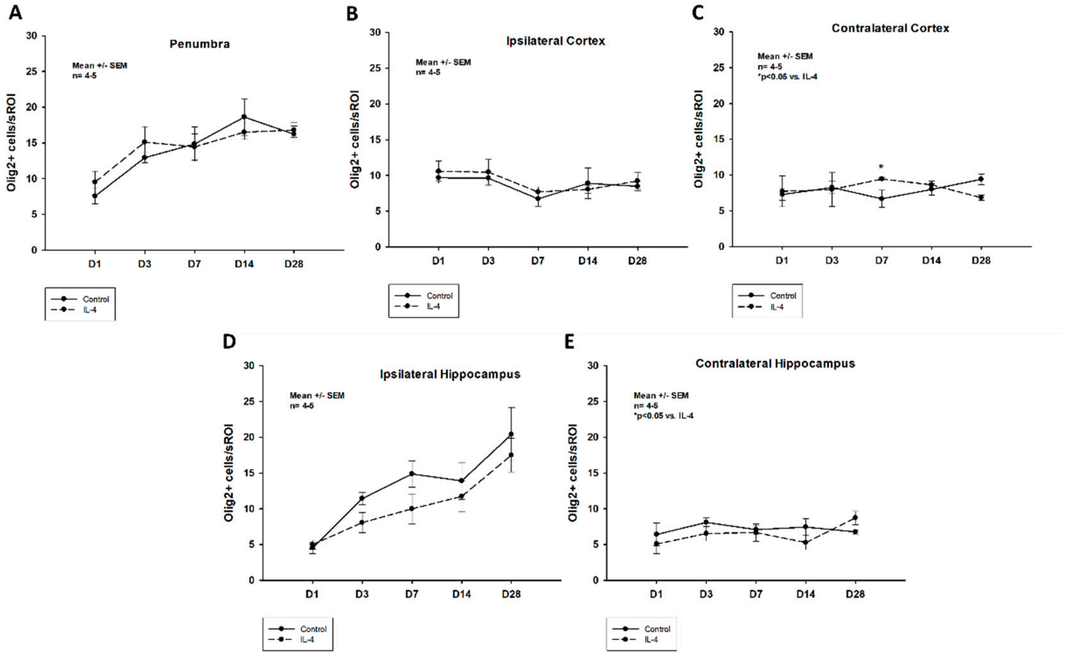

Supplement Figure S3. Number of Olig2+ cells in the penumbra (panel A), ipsi- and contralateral cortices (panels B and C) as well as ipsi- and contralateral hippocampi (panels D and E) within the first four weeks after CCI. The exact number of animals analyzed per group are summarized in Supplement Table S1. SEM: standard error of the mean, IL-4: interleukin-4 treated mice.

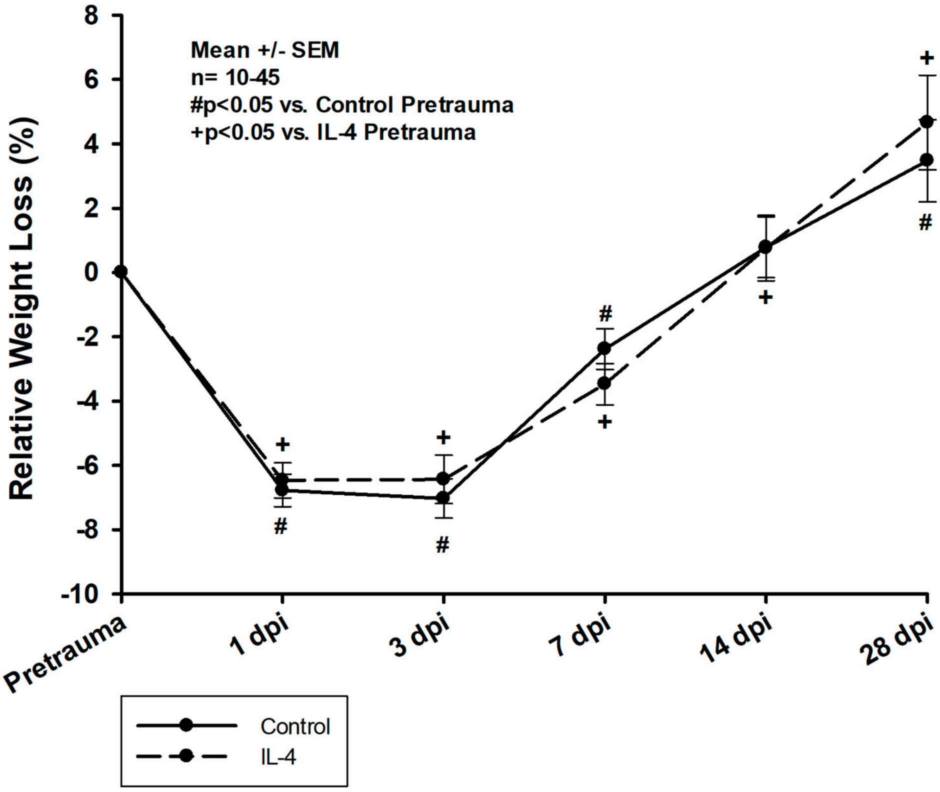

Supplement Figure S4. Relative weight loss within the first four weeks after CCI. The exact number of animals analyzed per group are summarized in Supplement Table S1. SEM: standard error of the mean, IL-4: interleukin-4 treated mice, dpi: days post injury.

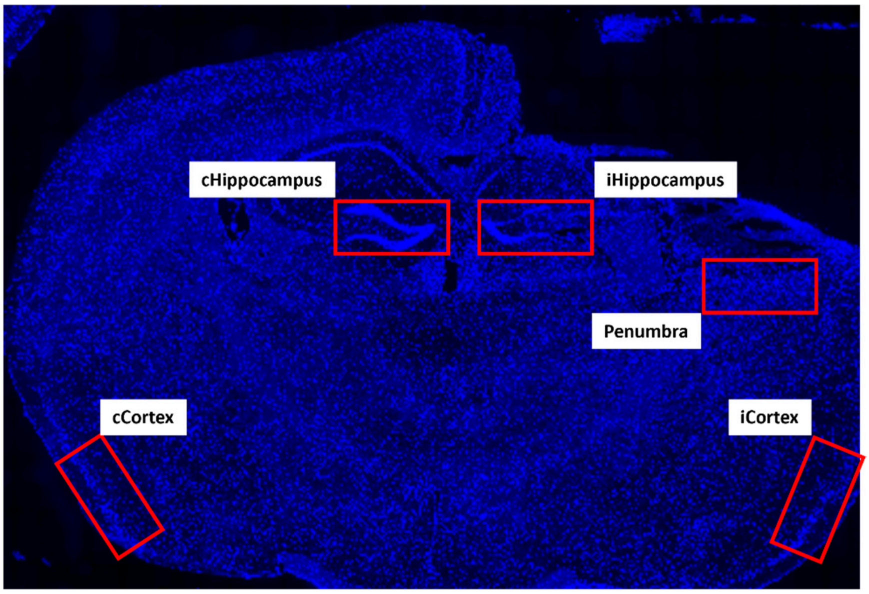

Supplement Figure S5. Coronal brain section displaying the five standardized regions of interest (sROI) consisting of 10000 pixels: ipsi- and cotralateral hippocampus, ipsi- and contralateral cortex and the traumatic penumbra.

	**Experimental Group**									
	Control 1dpi	*Control 3dpi*	*Control 7dpi*	*Control 14dpi*	*Control 28dpi*	*IL-4 1dpi*	*IL-4 3dpi*	*IL-4 7dpi*	*IL-4 14dpi*	*IL-4 28dpi*
**Histology**										
Lesion volume	7	7	8	8	8	7	5	6	8	8
Iba1+/CD86+ (all ROIs)	4	4	3	4	5	3	5	3	5	5
Iba1+/CD206+ (all ROIs)	4	4	3	4	5	3	5	3	5	5
GFAP (Penumbra)	5	5	4	5	5	5	5	4	5	5
GFAP (remaining ROIs)	5	5	5	5	5	5	5	5	5	5
NeuN+/TUNEL+ (Penumbra)	4*	5	5	5	5	5	5	5	4*	4*
NeuN+/TUNEL+ (iCortex)	5	5	4*	5	5	4*	5	5	4*	5
NeuN+/TUNEL+ (iHippocampus)	5	5	5	4	5	5	5	4	5	4
NeuN+/TUNEL+ (remaining ROIs)	5	5	5	5	5	5	5	5	5	5
MBP (Penumbra)	5	4	5	4	5	5	5	5	4*	5
MBP (iCortex)	5	4	5	3	5	5	5	5	5	5
MBP (cCortex)	5	4	5	4	5	5	5	5	5	5
MBP (iHippocampus)	5	4	4	4	5	5	5	4	5	5
MBP (cHippocampus)	5	4	5	4	5	5	5	5	5	5
Olig2+ (Penumbra)	5	5	4	5	4*	5	4	5	5	4
Olig2+ (iCortex)	5	5	4	5	5	5	4	5	5	5
Olig2+ (cCortex)	5	5	4	5	5	5	5	5	5	5
Olig2+ (iHippocampus)	5	5	4	5	5	5	5	4	5	5
Olig2+ (cHippocampus)	5	5	5	5	5	5	5	5	5	5
**Functional Tests**										
Weight	45	37	30	20	10	41	33	26	19	10
Hole Board	41	33*	26	17	8	39	31	24	17	9
Open Field	45	37*	30	20	10	41	33	26	19	10
CatWalkXT® Max Intensity (LF + LH + RH)	42	35*	30	18*	10	39	32*	26	18*	10
CatWalkXT® Max Intensity (RF)	36	36	30	20	10	38	36	26	17	9
Run Duration	42	37	30	20	10	39	33	30	19	10

Table S1 : Respective numbers of animals analyzed per group and parameter.
*unplausible outliers have been excluded, ROI: Region of interest, dpi: days post injury, iCortex: ipsilateral cortex, cCortex: contralateral cortex, iHippocampus: ipsilateral hippocampus, cHippocampus: contralateral hippocampus, LF: left forepaw, LH: left hindpaw, RF: right forepaw, RH: right hindpaw
